# False negative rate of COVID-19 PCR testing: a discordant testing analysis

**DOI:** 10.1186/s12985-021-01489-0

**Published:** 2021-01-09

**Authors:** Jamil N. Kanji, Nathan Zelyas, Clayton MacDonald, Kanti Pabbaraju, Muhammad Naeem Khan, Abhaya Prasad, Jia Hu, Mathew Diggle, Byron M. Berenger, Graham Tipples

**Affiliations:** 1 Public Health Laboratory, Alberta Precision Laboratories, University of Alberta Hospital, 8440 – 112 Street, Edmonton, AB T6G 2B7 Canada; 2 Division of Infectious Diseases, Department of Medicine, University of Alberta, Room 1NW-29, 16940 – 87 Avenue NW, Edmonton, AB T5R 4H5 Canada; 3 Department of Laboratory Medicine and Pathology, University of Alberta, University of Alberta Hospital, 8440 – 112 Street, Edmonton, AB T6G 2B7 Canada; 4 Division of Medical Microbiology and Infection Control, Vancouver Coastal Health Vancouver General Hospital, 899 W 12th Avenue, Vancouver, BC V5Z 1M9 Canada; 5 Public Health Laboratory, Alberta Precision Laboratories, Foothills Hospital, 1403 – 29 Street NW, Calgary, AB T2N 2T9 Canada; 6 Health Protection and Communicable Disease Control, Public Health, Alberta Health Services, Coronation Plaza, 14310 – 111 Avenue NW, Edmonton, AB T5M 3Z7 Canada; 7 Medical Officer of Health (MOH), Public Health, Alberta Health Services, 1213 – 4 Street SW, Calgary, AB T2R 0X7 Canada; 8 Department of Community Health Sciences, Cumming School of Medicine, University of Calgary, 1403 – 29 Street NW, Calgary, AB T2N 2T9 Canada; 9 Department of Pathology and Laboratory Medicine, University of Calgary, 3535 Research Road NW, Calgary, AB T2L 2K8 Canada; 10 Department of Medical Microbiology and Immunology, University of Alberta, 8440 – 112 Street, Edmonton, AB T6G 2B7 Canada; 11 Li Ka Shing Institute of Virology, University of Alberta, 6-010 Katz Group Centre for Pharmacy and Research, Edmonton, AB T6G 2E1 Canada

**Keywords:** SARS-CoV-2, COVID-19, Discordant testing, False negative rate

## Abstract

**Background:**

COVID-19 is diagnosed via detection of SARS-CoV-2 RNA using real time reverse-transcriptase polymerase chain reaction (rtRT-PCR). Performance of many SARS-CoV-2 rtRT-PCR assays is not entirely known due to the lack of a gold standard. We sought to evaluate the false negative rate (FNR) and sensitivity of our laboratory-developed SARS-CoV-2 rtRT-PCR targeting the envelope (E) and RNA-dependent RNA-polymerase (RdRp) genes.

**Methods:**

SARS-CoV-2 rtRT-PCR results at the Public Health Laboratory (Alberta, Canada) from January 21 to April 18, 2020 were reviewed to identify patients with an initial negative rtRT-PCR followed by a positive result on repeat testing within 14 days (defined as discordant results). Negative samples from these discordant specimens were re-tested using three alternate rtRT-PCR assays (targeting the E gene and N1/N2 regions of the nucleocapsid genes) to assess for false negative (FN) results.

**Results:**

During the time period specified, 95,919 patients (100,001 samples) were tested for SARS-CoV-2. Of these, 49 patients were found to have discordant results including 49 positive and 52 negative swabs. Repeat testing of 52 negative swabs found five FNs (from five separate patients). Assuming 100% specificity of the diagnostic assay, the FNR and sensitivity in this group of patients with discordant testing was 9.3% (95% CI 1.5–17.0%) and 90.7% (95% CI 82.6–98.9%) respectively.

**Conclusions:**

Studies to understand the FNR of routinely used assays are important to confirm adequate clinical performance. In this study, most FN results were due to low amounts of SARS-CoV-2 virus concentrations in patients with multiple specimens collected during different stages of infection. Post-test clinical evaluation of each patient is advised to ensure that rtRT-PCR results are not the only factor in excluding COVID-19.

## Background

Accurate case detection with rapid isolation and contact tracing form critical elements of the public health response to COVID-19. With most emerging infections, initially available nucleic acid tests (NATs) may lack data on the frequency of false negative results which can unnecessarily lead to repeated testing.

Studies of false-negative (FN) results from respiratory samples for SARS-CoV-2 are variable demonstrating FN rates (FNRs) ranging from 1 to 30% [1, 2]. FN results can occur for numerous reasons including suboptimal specimen collection, testing too early in the disease process, low analytic sensitivity, inappropriate specimen type, low viral load, or variability in viral shedding [3–9].

Implications of FN results can be significant, potentially leading to positive case clusters and negative outcomes [10]. Current guidance from the World Health Organization (WHO) and others calls for repeat testing (including sampling of the lower respiratory tract) in individuals who continue to display symptoms of COVID-19 with continued infection prevention measures [9, 11, 12]. The optimal interval of repeat testing is not clear with different studies suggesting a range from 1 to 6 days following the first negative test [13, 14].

The current study was designed to assess the FNR and sensitivity for the laboratory-developed test rtRT-PCR (LDT) used for frontline SARS-CoV-2 testing in Alberta, Canada, by determining the number of FN results in patients with repeat specimens submitted.

## Methods

### Setting, patients, and clinical samples

In the province of Alberta, Canada (population 4.4 million people), SARS-CoV-2 testing was conducted exclusively at the provincial Public Health Laboratory for symptomatic patients during the first four months of the pandemic [15–17]. The first case was confirmed on March 5, 2020 [18]. Test results and patient demographics were extracted from the laboratory information system to identify patients between January 21 and April 18, 2020, with an initial negative SARS-CoV-2 result followed by a positive result on repeat testing within 14 days (one incubation period) hereon defined as discordant test results [11].

Acceptable specimens for SARS-CoV-2 testing included nasopharyngeal (NP), oropharyngeal (OP), deep nasal turbinate swabs, endotracheal aspirates, and bronchoalveolar lavages (see Additional file 1: Table S1). All collection kits were internally validated prior to use.

### SARS-CoV-2 RNA detection

Nucleic acid extraction was performed on one of several platforms (see Additional file 1: Table S1). A LDT rtRT-PCR targeting the envelope (E) and RNA-dependent RNA-polymerase (RdRp) genes was used to detect SARS-CoV-2 RNA [19]. Samples with cycle threshold (Ct) values > 35 cycles were repeated in duplicate and considered positive if ≥ 2 of three results had an amplification curve. Invalid was used to refer to samples with PCR run errors such as instrument or internal control failure. The assay parameters and comparison to other assays used across Canada has been published [19, 20].

The negative samples from sets of discordant specimens were re-tested by rtRT-PCR for SARS-CoV-2 to evaluate for FNs. This was carried out by extracting nucleic acid from the original sample followed by testing using assays targeting three different genes: the E gene (using only the E gene target from the LDT in a singleplex format) and the N1/N2 portions of the nucleocapsid gene (see Additional file 1: Table S1) [21]. Evaluation of the CDC N1/N2 assay compared to the LDT demonstrated 94% positive agreement (95% CI 87.7–100%) and 100% negative agreement (see Additional file 2: Table S2).

The discordant samples were retrieved from storage at − 70 °C and underwent one freeze–thaw cycle. Samples that had tested positive were assumed to be true positives (based on the validation study of the LDT assay demonstrating analytic specificity of 100%) [19]. A negative sample was considered to be a FN if repeat testing yielded a positive result for ≥ 2 of three gene targets (E gene, N1, and/or N2).

### Evaluation of discordant swab quality

All swab sets identified as discordant were tested for the presence of human ribonuclease P (RNAse P) using an RT-PCR assay (see Additional file 1: Table S1) [21].

### Statistical analysis

Statistical comparison of parametric variables was done using independent t-tests and non-parametric variables using the Wilcoxon matched-pairs signed rank test. Data analyses were conducted in Stata 14.2 software (Statacorp LP, 2015, College Station, USA).

## Results

Between January 21 and April 18, 2020, 100,001 COVID-19 tests (95,919 patients) were completed with 1954 (2%) individual cases confirmed (see Additional file 3: Figure S1). Including repeat tests, the overall positivity rate was 2.2%.

Forty-nine (0.05%) were found to have discordant results (total 101 swabs including 46 patients with two swabs and 3 patients with three swabs). The median age of these patients was 72 years (range 25–97) with 69.4% being female and 26.5% requiring hospitalization (Table 1).

**Table 1:** Demographic characteristics of 49 patients with discordant swab results for COVID-19

Variable		%
Age (years)		
Median	72	
Range	25–97	
Sex		
Male	15	30.6
Female	34	69.4
Exposure to a known case		
Yes	38	77.6
Acquisition		
Health care	9	18.4
Community	38	77.6
Unknown	2	4.0
Healthcare worker	7	14.3
Hospitalization	13	26.5
Travel history	4	8.2

All 101 discordant swabs were available for further evaluation (herein identified as swab 1, swab 2, and swab 3) (Table 2). Original testing results of these 49 patients showed: swab 1 for all 49 patients was negative; swab 2 for 46/49 patients was positive, and swab 3 was positive for 3/3 patients. Repeat testing of swab 1 for each of the 49 patients using a combination of three alternate assays revealed five FN results (Table 2). Of these, 3/5 were NP swabs in UTM and 2/5 were Aptima® swabs used for deep nasal sampling. Ct values for repeat testing of swab 1 specimens among the three different assays ranged from 32.7 to 38.8 cycles (median 35.5). Five swab 1 specimens re-tested positive on the E gene assay and the CDC N2 assay; two swab 1 specimens re-tested positive by all three alternate assays. The mean times of collection (in days) between swab 1 and swab 2 for the FN and non-FN discrepant specimens were 6.1 (*p* = 0.06) and 3.3 (*p* = 0.20), respectively.

**Table 2:** Evaluation of 49 patients (101 swabs) with discordant COVID-19 testing and confirmatory testing results

	Swab 1 (n = 49 swabs)	Swab 2 (n = 49 swabs)	Swab 3 (n = 3 swabs)^c^
NP swab in UTM (%)	29 (59.2)	32 (65.3)	2 (66.7)
Deep nasal turbinate swab (%)	20 (40.8)	9 (18.4)	1 (33.3)
Oropharyngeal swab (%)	0	8 (16.3)	0
Original swab test result^a^ (E gene Ct median; range) (RdRp gene Ct median; range)	49 negative	46 positive 3 negative (19.5; 13.0–35.5) (22.4; 16.2–37.8)	3 positive 0 negative (13.9; 12.5–24.7) (16.5; 14.8–28.0)
Positive result on E gene assay^b^ (Ct range)	5/49 (34.1–37.9)	0/3	ND
Positive result on CDC N1 assay^b^ (Ct range)	2/49 (32.7–35.9)	0/3	ND
Positive result on CDC N2 assay^b^ (Ct range)	5/49 (33.2–38.8)	0/3	ND

*Ct* cycle threshold (cycles), *E* envelope, *LDT* laboratory developed test, *ND* not done, *NP* nasopharyngeal, *RdRp* RNA dependent RNA polymerase, *UTM* universal transport media

^a^Testing done using E gene/RdRp gene LDT SARS-CoV-2 rtRT-PCR

^b^Repeat testing conducted only on negative swabs to evaluate for false-negative results

^c^Only 3 of 49 patients had a third swab done

No significant differences in the Ct values for human RNAse P were noted between swabs 1, 2, and 3 (see Additional file 4: Figure S2; all *p*-values > 0.05).

From the five FN specimens, 4/5 had swab 1 collected on or the day after date of symptom onset (DSO) (Table 3). The maximum duration between DSO and swab 1 was 9 days and swab 2 was eleven days. Swab 2 for all five patients was collected post-DSO (4–11 days). All patients with FN results had community-acquired SARS-CoV-2 infection; three were healthcare workers and three had exposure to a confirmed COVID-19 case.

**Table 3:** Timeline (in days) of swab collection and epidemiologic information of five false negative discordant specimens

Patient	− 5	− 4	− 3	− 2	− 1	0	1	2	3	4	5	6	7	8	9	10	11	Exposed to confirmed case?	Healthcare worker?	Community- or healthcare-acquired?
1						DSO	S1			S2								Yes	Yes	Community-acquired
2						DSO		S1				S2						No	Yes	Community-acquired
3						DSO									S1		S2	Yes	No	Community-acquired
4						DSO, S1						S2						Unknown	No	Community-acquired
5	S1					DSO						S2						Yes	Yes	Community-acquired

Negative numbers indicate days before DSO; day 0 indicates DSO; positive numbers indicate days post-DSO

*DSO* date of symptom onset, *S1* swab 1, *S2* swab 2

Based on the additional testing conducted, 5/101 negative swabs were considered FNs with 49/101 presumed to be true positives (TPs). Therefore, FNR (FN/[FN + TP]) in this subset of patients with discordant swabs is 9.3% (95% CI 1.5–17.0%). By extension, the sensitivity (1-FNR) of testing in this subset of discordant swabs is 90.7% (95% CI 82.6–98.9%).

## Discussion

The major strength of this study lies in the large sample size (100,001 SARS-CoV-2 rtRT-PCR tests from 95,919 patients) from which discordant results were identified. Discordant results were found for 0.05% of all patients tested. Based on re-testing of 49 patients with discordant results, the FNR and sensitivity of our LDT in this subgroup of patients was approximately 9.3% and 90.7%, respectively.

The FNR calculated from our data analysis is comparable to other reports. Data from earlier in the pandemic reported FNRs of up to 30% [6] with a systematic review on the topic reporting ranges from 2 to 29% [2]. A large study from New York evaluating the clinical performance of SARS-CoV-2 molecular testing found that on average up to 17% of positives were missed by the first test [22], while another American study reported a FNR of 3.5% in patients with discordant swab results within a 7-day period [1]. Two other studies have estimated sensitivities ranging from 89 to 94.6% [22, 23].

In our study, specimen quality was not considered a contributing factor given human DNA content did not differ significantly across all the swabs. A similar approach using RNase P as a surrogate for quality of swab collection has been used in several other studies [3, 23, 24].

The five FNs were likely caused by changes in viral load and shedding over time. Based on Ct values, all FNs were found to have low levels of viral RNA. Four of five FN samples had early collections related to the DSO (from 5 days prior to symptom onset to 2 days post-symptom onset). The other FN sample was collected 9 days post-symptom onset with the swab found to be positive for this patient with routine testing having been collected 2 days later, which could be related to variable shedding after the acute phase of infection [8]. Variable shedding dynamics have also been noted by authors of a pooled analysis of 1330 samples with FNR estimated as 20% at three days post DSO, 38% on the DSO, and 67% on the day prior to DSO [4].

Three of five FN swabs were collected using an NP flocked swab in UTM and the other two were collected using the Aptima® swab and transport medium. While this may indicate that these swab types and media did not influence the FNR, more data is needed to support this. However, one study indicated that Aptima® products are as good or better than routine flocked NP/UTM swabs for detecting SARS-CoV-2, attributed in part to the preservatives in the Aptima® transport solution preventing RNA degradation [25].

The principal limitations of this study are its retrospective nature and that FN samples were biased towards patients undergoing repeat swab collection, likely due to high suspicion of COVID-19. Ideally, a cohort of negative patients would be tested using multiple NAT tests and re-tested prospectively, but this poses logistical challenges and would require a large number of patients to be screened. Another limitation is the assumption that all positives by the local LDT were true positives. However, the analytical specificity of the LDT is reported as 100% [19] and it demonstrated a high negative percent agreement with the CDC N1/N2 assay. Most other SARS-CoV-2 rtRT-PCR assays have shown high clinical specificities, making this a reasonable assumption [26, 27].

## Conclusions

This work adds to the literature by demonstrating that the FNR of SARS-CoV-2 molecular assays is low [1, 27, 28] and subject to viral load dynamics over time. However, the interpretation of COVID-19 test results should be conducted in the overall context of each patient’s clinical presentation [9, 29], with repeat testing advised should post-test probability upon follow-up clinical evaluation remain high.

## Supplementary information

**Additional file 1: Table S1.** Commercial products utilised in the process of SARS-CoV-2 rtRT-PCR testing.

**Additional file 2: Table S2.** Comparison of LDT to CDC N1/N2 assay using 100 specimens selected randomly from those tested.

**Additional file 3: Figure S1.** Results of COVID-19 testing from the first 100,001 tests completed.

**Additional file 4: Figure S2.** RNAse P detection (based on Ct value) between swab sets **(a)** on 46 patients who had two swabs that were discordant; and **(b)** on 3 patients with three swabs.

## Data Availability

The datasets used and/or analysed during the current study are available from the corresponding author on reasonable request.

## Abbreviations

Ct: Cycle threshold
DSO: Date of symptom onset
FN: False-negative
FNR: False-negative rate
LDT: Laboratory developed test
NATs: Nucleic acid tests
NP: Nasopharyngeal
OP: Oropharyngeal
PCR: Polymerase chain reaction
RdRp: RNA-dependent RNA-polymerase
RNAse P: Human ribonuclease P
rtRT-PCR: Real-time reverse-transcriptase polymerase chain reaction
UTM: Universal transport media
WHO: World Health Organization
