# Supplementary material for: False negative rate of COVID-19 PCR testing: a discordant testing analysis
Source: Virol J. 2021 Jan 9;18:13. doi: 10.1186/s12985-021-01489-0 (PMC7794619; doi:10.1186/s12985-021-01489-0)
Supplement: Supplementary file 1 — Additional file 1: Table S1. Commercial products utilised in the process of SARS-CoV-2 rtRT-PCR testing. [file 12985_2021_1489_MOESM1_ESM.docx]

**Additional file 1: Table S1.** Commercial products utilised in the process of SARS-CoV-2 rtRT-PCR testing.

| Component of SARS-CoV-2 rtRT-PCR testing | Product (Use) | Company |
| --- | --- | --- |
| Swabs and transport media | Mini-tip flocked swabs with universal transport media (UTM) (NP swabs) | Copan Diagnostics, Bresica, Italy |
|  | Aptima Unisex collection kits (deep nasal turbinate sampling) | Hologic, Marlborough, USA |
|  | Aptima Multitest collection kit (throat sampling) | Hologic, Marlborough, USA |
| Nucleic acid extraction platforms | Microlab STARlet | Hamilton Company, Reno, USA |
|  | NucliSENS EasyMAG | BioMérieux, Marcy-L’Etoile, France |
|  | MagMAX | ThermoFisher Scientific, Waltham, USA |
| PCR Kit | Centers for Disease Control (Atlanta, USA) SARS-CoV-2 assay | Integrated DNA Technologies, Coralville, USA |
|  | RNAse P rtRT-PCR kit | Integrated DNA Technologies, Coralville, USA |
