# Supplementary material for: False negative rate of COVID-19 PCR testing: a discordant testing analysis
Source: Virol J. 2021 Jan 9;18:13. doi: 10.1186/s12985-021-01489-0 (PMC7794619; doi:10.1186/s12985-021-01489-0)
Supplement: Supplementary file 2 — Additional file 2: Table S2. Comparison of LDT to CDC N1/N2 assay using 100 specimens selected randomly from those tested. [file 12985_2021_1489_MOESM2_ESM.docx]

**Additional file 2: Table S2.** Comparison of LDT to CDC N1/N2 assay using 100 specimens selected randomly from those tested.

|  |  | Result on CDC N1/N2 multiplex assay* | | | |
| --- | --- | --- | --- | --- | --- |
|  |  | N1 positive / N2 negative | N1 negative / N2 positive | N1 + N2 positive | N1 + N2 negative |
| LDT result | Positive  (n = 51) | 1 | 2 | 48 | 0 |
|  | Negative  (n = 49) | 0 | 0 | 0 | 49 |

*CDC assay results were considered positive when both N1 and N2 targets were positive. Specimens were deemed inconclusive if only one of the N1 or N2 targets tested positive.
