# Supplementary material for: False negative rate of COVID-19 PCR testing: a discordant testing analysis
Source: Virol J. 2021 Jan 9;18:13. doi: 10.1186/s12985-021-01489-0 (PMC7794619; doi:10.1186/s12985-021-01489-0)
Supplement: Supplementary file 3 — Additional file 3: Figure S1. Results of COVID-19 testing from the first 100,001 tests completed. [file 12985_2021_1489_MOESM3_ESM.docx]

**Additional file 3: Figure S1.** Results of COVID-19 testing from the first 100,001 tests completed.

100,001 tests performed

- 95,919 patients
- 2,213 positive tests
- 97,744 negative tests
- 44 invalid tests

Single test performed

- 92,486 patients and tests
- 1,690 positive tests (1.8%)
- 90,783 negative tests (98.2%)
- 13 invalid tests (0.01%)

Repeat tests performed

- 3,433 patients
- 7,515 tests
- 523 positive tests
- 6,961 negative tests
- 31 invalid tests

First test negative

- 3,223 patients
- 6,879 tests
- 77 positive tests
- 6,797 negative tests
- 5 invalid tests

First test invalid

- 15 patients
- 41 tests
- 0 positive tests
- 19 negative tests
- 22 invalid tests

First test positive

- 195 patients
- 595 tests
- 446 positive tests
- 145 negative tests
- 4 invalid tests

All repeat tests negative or invalid

- 3,154 patients
- 6,725 tests
- 0 positive tests
- 6,720 negative tests
- 5 invalid tests

At least one repeat positive (discordant)*

- 69 patients
- 154 tests
- 77 positive tests
- 77 negative tests
- 0 invalid tests

*49 patients met the definition of discordant within 14 days:

- 101 tests
- 49 positive tests
- 52 negative tests
- 0 invalid tests
