# Supplementary material for: False negative rate of COVID-19 PCR testing: a discordant testing analysis
Source: Virol J. 2021 Jan 9;18:13. doi: 10.1186/s12985-021-01489-0 (PMC7794619; doi:10.1186/s12985-021-01489-0)
Supplement: Supplementary file 4 — Additional file 4: Figure S2. RNAse P detection (based on Ct value) between swab sets (a) on 46 patients who had two swabs that were discordant; and (b) on 3 patients with three swabs. [file 12985_2021_1489_MOESM4_ESM.docx]

A

**Additional file 4: Figure S2.** RNAse P detection (based on Ct value) between swab sets **(a)** on 46 patients who had two swabs that were discordant; and **(b)** on 3 patients with three swabs.
